# Increased accumulation of doxorubicin and doxorubicinol in cardiac tissue of mice lacking mdr1a P-glycoprotein

**DOI:** 10.1038/sj.bjc.6690019

**Published:** 1999-09-24

**Authors:** J van Asperen, O van Tellingen, F Tijssen, A H Schinkel, J H Beijnen

**Affiliations:** Department of Clinical Chemistry, The Netherlands Cancer Institute, Plesmanlaan 121, Amsterdam, 1066 CX, The Netherlands; Department of Experimental Therapy, The Netherlands Cancer Institute, Plesmanlaan 121, Amsterdam, 1066 CX, The Netherlands; Department of Pharmacy, Slotervaart Hospital, Louwesweg 6, Amsterdam, 1066 EC, The Netherlands

**Keywords:** P-glycoprotein, doxorubicin, reversal agents, cardiotoxicity, pharmacokinetics

## Abstract

To gain more insight into the pharmacological role of endogenous P-glycoprotein in the metabolism of the widely used substrate drug doxorubicin, we have studied the plasma pharmacokinetics, tissue distribution and excretion of this compound in *mdr1a(–/–* and wild-type mice. Doxorubicin was administered as an i.v. bolus injection at a dose level of 5 mg kg^−1^. Drug and metabolite concentrations were determined in plasma, tissues, urine and faeces by high-performance liquid chromatography. In comparison with wild-type mice, the terminal half-life and the area under the plasma concentration–time curve of doxorubicin in it>mdr1a(–/–) mice were 1.6- and 1.2-fold higher respectively.The retention of both doxorubicin and its metabolite doxorubicinol in the hearts of *mdr1a(–/–)* mice was substantially prolonged. In addition, a significantly increased drug accumulation was observed in the brain and the liver of *mdr1a(–/–)* mice. The relative accumulation in most other tissues was not or only slightly increased. The differences in cumulative faecal and urinary excretion of doxorubicin and metabolites between both types of mice were small. These experiments demonstrate that the absence of mdr1a P-glycoprotein only slightly alters the plasma pharmacokinetics of oxorubicin. Furthermore, the substantially prolonged presence of both doxorubicin and doxorubicinol in cardiac tissue of *mdr1a(–/–)* mice suggests that a blockade of endogenous P-glycoprotein in patients, for example by a reversal agent, may enhance the risk of cardiotoxicity upon administration of doxorubicin. © 1999 Cancer Research Campaign


British Journal of Cancer (1999) **79**(1), 108–113
© 1999 Cancer Research Campaign

### Increased accumulation of doxorubicin and doxorubicinol in cardiac tissue of mice lacking mdr1a P-glycoprotein

**J van Asperen[1], O van Tellingen[1], F Tijssen[1], AH Schinkel[2] and JH Beijnen[1,3]**

[1]Department of Clinical Chemistry, The Netherlands Cancer Institute, Plesmanlaan 121, 1066 CX Amsterdam, The Netherlands; [2]Department of Experimental Therapy, The Netherlands Cancer Institute, Plesmanlaan 121, 1066 CX Amsterdam, The Netherlands; [3]Department of Pharmacy, Slotervaart Hospital, Louwesweg 6, 1066 EC Amsterdam, The Netherlands

**Summary** To gain more insight into the pharmacological role of endogenous P-glycoprotein in the metabolism of the widely used substrate drug doxorubicin, we have studied the plasma pharmacokinetics, tissue distribution and excretion of this compound in *mdr1a(–/–)* and wild-type mice. Doxorubicin was administered as an i.v. bolus injection at a dose level of 5 mg $kg^{-1}$. Drug and metabolite concentrations were determined in plasma, tissues, urine and faeces by high-performance liquid chromatography. In comparison with wild-type mice, the terminal half-life and the area under the plasma concentration–time curve of doxorubicin in *mdr1a(–/–)* mice were 1.6- and 1.2-fold higher respectively. The retention of both doxorubicin and its metabolite doxorubicinol in the hearts of *mdr1a(–/–)* mice was substantially prolonged. In addition, a significantly increased drug accumulation was observed in the brain and the liver of *mdr1a(–/–)* mice. The relative accumulation in most other tissues was not or only slightly increased. The differences in cumulative faecal and urinary excretion of doxorubicin and metabolites between both types of mice were small. These experiments demonstrate that the absence of mdr1a P-glycoprotein only slightly alters the plasma pharmacokinetics of doxorubicin. Furthermore, the substantially prolonged presence of both doxorubicin and doxorubicinol in cardiac tissue of *mdr1a(–/–)* mice suggests that a blockade of endogenous P-glycoprotein in patients, for example by a reversal agent, may enhance the risk of cardiotoxicity upon administration of doxorubicin.

**Keywords:** P-glycoprotein; doxorubicin; reversal agents; cardiotoxicity; pharmacokinetics

P-glycoprotein is a large plasma membrane protein that can cause multidrug resistance in tumour cells by actively extruding substrate drugs out of the cell. These substrates include many anti-cancer drugs, such as *vinca* alkaloids, taxanes, epipodophyllotoxins and anthracyclines (reviewed in Endicott and Ling, 1989). The discovery that verapamil was able to reverse multidrug resistance in murine leukaemia cell lines (Tsuruo et al, 1981) initiated the search for reversal agents, which are compounds capable of blocking or inhibiting P-glycoprotein. A major concern for the clinical application of effective reversal agents are the potential consequences of inhibition of endogenous P-glycoprotein. To predict possible adverse effects of reversal agents and to gain more insight into the physiological role of endogenous P-glycoproteins, mice with homozygously disrupted P-glycoprotein genes have been generated at our institute (Schinkel et al, 1994, 1997).

In humans, only one P-glycoprotein (MDR1) plays a role in multidrug resistance, whereas in mice both mdr1a and mdr1b P-glycoproteins are involved. The tissue distribution of these proteins suggests that the two murine isoforms together perform the same function as the single human MDR1 protein. The *mdr1a* gene is predominantly expressed in the intestines and in the capillaries of the brain and the testis, *mdr1b* is mainly expressed in the adrenal gland, pregnant uterus and ovarium. Significant levels of both mdr1a and mdr1b P-glycoprotein are present in liver, kidney, lung, heart and spleen (Cordon-Cardo et al, 1989; Croop et al, 1989). Based on the results of tissue distribution studies, it has been suggested that P-glycoprotein plays a role in the protection of the organism against potentially toxic agents, e.g. by limiting the absorption of orally ingested compounds, by mediating the elimination of substrates from the body and by protecting essential organs such as the brain and the testis against toxic substances in the circulation (Thiebaut et al, 1987; Cordon-Cardo et al, 1989). Recent studies confirmed that P-glycoprotein in the blood–brain barrier protects the brain against the entry of toxic compounds, whereas P-glycoprotein in the intestinal epithelium has been shown to limit the uptake of substrates from the intestinal lumen and to mediate their direct excretion from the bloodstream (Schinkel et al, 1994, 1995, 1996; Mayer et al, 1996; Sparreboom et al, 1997).

To gain a detailed insight into the pharmacokinetic consequences of blocking P-glycoprotein in normal tissues, we previously performed a comprehensive analysis of the plasma pharmacokinetics, tissue distribution and excretion of vinblastine and its metabolites in wild-type and *mdr1a(–/–)* mice (Van Asperen et al, 1996). However, it is of importance to obtain also comparable data on other widely used substrate drugs because it is likely that the impact of endogenous P-glycoprotein on the pharmacokinetics is substrate dependent. Here, we report on the comparative pharmacokinetics of doxorubicin and metabolites in wild-type and *mdr1a(–/–)* mice.

*Received 5 February 1998*
*Revised 15 May 1998*
*Accepted 21 May 1998*

*Correspondence to:* J van Asperen, Drug Metabolism Department, Covance Laboratories Ltd., Otley Road, Harrogate, North Yorkshire HG3 1PY, UK

### MATERIALS AND METHODS

### Animals

Female FVB wild-type and *mdr1a(–/–)* mice between 10 and 16 weeks of age were used in all experiments. The animals were housed and handled according to institutional guidelines. Food (Hope Farms, Woerden, The Netherlands) and acidified water were given ad libitum.

### Drugs and chemicals

Doxorubicin hydrochloride (Adriblastina) and daunorubicin hydrochloride (Cerubidin) were purchased as powder for injection from Pharmacia-Farmitalia-Carlo Erba (Milan, Italy) and Rhône-Poulenc Rorer (Antony Cedex, France) respectively. A solution for injection was prepared by dissolving 10 mg of doxorubicin hydrochloride in 5 ml of saline (NPBI, Emmer-Compascuum, The Netherlands), yielding a final concentration of 2 mg $ml^{-1}$. The metabolites doxorubicinol, 7-deoxydoxorubicinolone and 7-deoxydoxorubicinone were kindly provided by Pharmacia-Farmitalia-Carlo Erba. Bovine serum albumin (BSA) was obtained from Organon Teknika (Boxtel, The Netherlands). All other reagents were of analytical or Lichrosolv gradient grade and were purchased from E Merck (Darmstadt, Germany). Water was purified by the Milli-Q Plus system (Millipore, Milford, USA). Blank human plasma was obtained from healthy donors.

### Study design

Doxorubicin was administered as an i.v. bolus injection via a tail vein of diethyl ether-anaesthetized mice at a dose level of 5 mg $kg^{-1}$ body weight. The excretion study was performed with two groups of six animals, whereas in the other pharmacokinetic experiments three or four animals were used per time point. Tissue and blood samples were taken at 1, 4 and 24 h after drug administration. Blood was obtained by orbital bleeding under diethyl ether anaesthesia and collected in heparinized tubes. The animals were sacrificed by cervical dislocation to collect the following tissues: brain, skeletal muscle, colon, caecum, small gut, stomach, liver (without gall bladder), kidney, lung, spleen, heart, ovary, uterus and breast. The tissues were homogenized with a Polytron tissue homogenizer (Kinematica, Littau, Switzerland) in 4% (w/v) BSA in water, resulting in final concentrations of approximately 0.05–0.2 g tissue $ml^{-1}$. Additional blood samples were obtained at 5, 15, 30 and 45 min and at 2, 8, 16, 30, 40, 56 and 64 h after drug administration. Blood samples were centrifuged (10 min, 2000 ***g***, 4°C) to separate the plasma fraction, which was stored for analysis. Urine and faeces from mice kept in Ruco Type M/1 stainless-steel metabolic cages (Valkenswaard, The Netherlands) were collected during time intervals of 0–8, 8–24, 24–48, 48–72 and 72–96 h after drug administration. Homogenization of faeces in 4% (w/v) BSA in water (0.03–0.1 g faeces $ml^{-1}$) was according to the procedure described above for tissue specimens. All biological specimens were stored at –20°C until analysis.

### Drug analysis

Doxorubicin and its metabolites doxorubicinol, 7-deoxydoxorubicinolone and 7-deoxydoxorubicinone were quantified in plasma, tissues, urine and faeces according to a validated high-performance liquid chromatographic (HPLC) method, which was shown to be suitable for comparative pharmacokinetic studies (Van Asperen et al, 1998). In brief, dilutions of faeces homogenate (20-fold) and urine (100-fold) were prepared in blank human plasma, all other specimens were used without further dilution. Sample volumes of 50–200 μl were used, aliquots < 200 μl were supplemented with blank human plasma to a final volume of 200 μl. Next, 100 μl of a solution of the internal standard daunorubicin and 200 μl of a 6% borate buffer (pH 9.5) were added. The analytes were extracted from the samples with 1 ml of chloroform-1-propanol (4:1, v/v) by mixing for 5 min. After centrifugation for 10 min at 4°C (3000 ***g***), the aqueous layer and the pellet were removed by suction. The organic layer was evaporated in vacuo in a Speed-Vac Plus SC210A system (Savant, Farmingdale, USA) at 43°C. The residue was reconstituted in 100 μl of acetonitrile–tetrahydrofuran (40:1, v/v) by sonication for 5 min. After adding 300 μl acidified water (pH 2.05) and vortexing, a 50-μl aliquot was injected into the HPLC system. The reversed-phase chromatographic system consisted of a SpectroFlow 400 solvent delivery system (Kratos, Ramsey, USA), a Basic Marathon autosampler provided with a cooled (4°C) sample tray (Spark Holland, Emmen, The Netherlands) and a Model FP920 fluorescence detector (Jasco, Hachioji City, Japan). Doxorubicin and metabolites were separated at ambient temperature using a Chromsep glass analytical column (100 × 3 μm, ID) packed with 7-μm Lichrosorb RP-8 material (Chrompack, Middelburg, The Netherlands). The mobile phase consisted of acidified water (pH 2.05)–acetonitrile–tetrahydrofuran (80:30:1, v/v/v) and was delivered at a flow rate of 0.4 ml $min^{-1}$. The column eluent was monitored fluorimetrically at an excitation wavelength of 460 nm and an emission wavelength of 550 nm, with a bandwidth of 40 nm. Peak recording and integration were performed with an SP4600 DataJet integrator connected to a WINner/286 data station (Spectra Physics, San Jose, USA). Calibration curves, prepared in blank human plasma, were calculated by weighted ($1/y^2$) least squares linear-regression analysis of the nominal concentration (abscissa) versus the ratio ($y$) of the peak area of each of the compounds and the internal standard (ordinate).

**Table 1** Pharmacokinetic parameters of doxorubicin in wild-type and *mdr1a(–/–)* mice after i.v. bolus administration of 5 mg $kg^{-1}$

| Parameter | Wild-type mice | *mdr1a(–/–)* mice |
|---|---|---|
| $AUC_{(0–64)}$ | 1818 ± 45 | 2269* ± 52 nmol.h $l^{-1}$ |
| Cl | 4.5 ± 0.1 | 3.6* ± 0.1 l $h^{-1}$ $kg^{-1}$ |
| $V_d$ | 101 ± 3.8 | 128* ± 5.5 l $kg^{-1}$ |
| $t_{1/2(\gamma)}$ | 15.7 ± 0.2 | 24.8* ± 0.4 h |

Data, means ± standard errors; $AUC_{(0–64)}$, area under the plasma concentration–time curve up to 64 h after drug administration; Cl, clearance; $t_{1/2(\gamma)}$, elimination half-life; *$P < 0.05$ vs wild-type mice.

### Pharmacokinetics

Pharmacokinetic parameters were calculated by non-compartmental methods using the software package Quattro Pro for Windows (Version 5.0, 1993; Borland International, Scotts Valley, USA). The area under the plasma concentration–time curve (AUC) was calculated by the linear trapezoidal rule without extrapolation to infinity. The standard error of the AUC was calculated with the law of propagation of errors. To calculate the body clearance (Cl), the following formula was used:

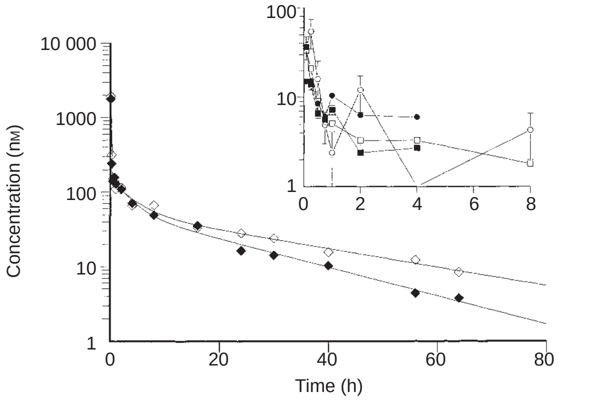

**Figure 1** Plasma concentration vs time curve of doxorubicin in *mdr1a(–/–)* (◇) and wild-type mice (◆) after i.v. bolus administration of 5 mg kg–1. Inset: plasma concentrations of doxorubicinol (squares) and 7-deoxydoxorubicinolone (circles) in *mdr1a(–/–)* (open symbols) and wild-type (closed symbols) mice. Data are shown as the mean concentrations, and error bars represent the standard errors. The absence of error bars indicates that the standard error is smaller than the size of the symbols

(1) Cl = dose/AUC

The elimination rate constant ($k$) and the standard error of $k$ were calculated by linear regression analysis of the ln(concentration) vs time data points of the final part of the plasma concentration–time curve. Subsequently, the terminal half-life ($t_{1/2(\gamma)}$) was calculated with the formula:

(2) $t_{1/2(\gamma)} = \ln 2/k$

The volume of distribution ($V_d$) was calculated using the formula:

(3) $V_d = \mathrm{Cl}/k$

### Statistics

The unpaired Student's $t$-test (two-tailed) was used to compare the pharmacokinetics of wild-type and *mdr1a(–/–)* mice. A $P$-value of less than 0.05 was regarded as significant.

### RESULTS

A limited toxicity study revealed that a 5 mg kg$^{-1}$ dose level of doxorubicin was well tolerated by both types of mice (data not shown). The plasma concentration–time curves of doxorubicin in wild-type and *mdr1a(–/–)* mice coincided, but diverged at approximately 16 h after drug administration because of a prolonged $t_{1/2(\gamma)}$ in *mdr1a(–/–)* mice (Figure 1 and Table 1). Consequently, a 1.2-fold higher AUC was observed in *mdr1a(–/–)* mice than in wild-type mice. Low plasma concentrations of all metabolites were detected in both types of mice, with 7-deoxydoxorubicinone only detectable at 5 min after drug administration. The irregular shape of the plasma concentration–time curve of 7-deoxydoxorubicinolone was in agreement with previous observations in humans (Mross et al, 1988). The $V_d$ of doxorubicin was 1.3-fold higher in *mdr1a(–/–)* mice than in wild-type mice.

An increased accumulation of doxorubicin in brain and liver of *mdr1a(–/–)* mice was observed at all time points (Table 2). In all other tissues, minor differences were found at 1 and 4 h after drug administration, whereas at 24 h relatively higher doxorubicin concentrations were observed in heart, intestinal tissues, lung and breast of *mdr1a(–/–)* mice. All metabolites were present in an organ-specific fashion, which was similar in both types of mice. The metabolites doxorubicinol and 7-deoxydoxorubicinolone were detected in all tissues, except for brain for doxorubicinol and brain, ovary and uterus for 7-deoxydoxorubicinolone. The metabolite 7-deoxydoxorubicinone was only found in caecum, small gut, liver, kidney and breast. An increased accumulation of all metabolites was observed in the liver of *mdr1a(–/–)* mice (Table 3). At 24 h after drug administration, the concentrations of doxorubicinol and 7-deoxydoxorubicinolone were also higher in most other tissues of *mdr1a(–/–)* mice, e.g. the heart and the kidney (Table 3). No clear differences were observed at 1 and 4 h. Of all organs, the liver and the kidney accumulated the highest amounts of each metabolite.

Over a period of 96 h after injection, 18% and 13% of the administered dose were recovered as doxorubicin plus metabolites in urine of *mdr1a(–/–)* and wild-type mice respectively. The cumulative faecal excretion of these compounds was below 13% of the dose in both types of mice (Table 4).

**Table 2** Tissue levels of doxorubicin in wild-type and *mdr1a(–/–)* mice at 1, 4 and 24 h after i.v. administration of 5 mg kg$^{-1}$

| | 1 h | | | 4 h | | | 24 h | | |
|---|---|---|---|---|---|---|---|---|---|
| | Wild-type | *mdr1a(–/–)* | Ratio | Wild-type | *mdr1a(–/–)* | Ratio | Wild-type | *mdr1a(–/–)* | Ratio |
| Plasma | 130 ± 18.5 | 110 ± 3.5 | 0.9 | 70.9 ± 6.12 | 66.2 ± 4.96 | 0.9 | 16.3 ± 0.39 | 28.2 ± 4.30 | 1.7 |
| Brain | 0.10 ± 0.01 | 0.28 ± 0.01 | 2.8 | 0.12 ± 0.02 | 0.33 ± 0.02 | 2.8 | 0.06 ± 0.01 | 0.21 ± 0.02 | 3.8 |
| Muscle | 6.95 ± 0.65 | 6.03 ± 0.61 | 0.9 | 7.92 ± 0.74 | 6.58 ± 0.60 | 0.8 | 1.35 ± 0.13 | 1.88 ± 0.14 | 1.4 |
| Colon | 8.17 ± 0.83 | 9.65 ± 0.78 | 1.2 | 13.0 ± 0.84 | 12.6 ± 1.33 | 1.0 | 2.99 ± 0.36 | 7.70 ± 0.25 | 2.6 |
| Caecum | 7.95 ± 0.04 | 9.64 ± 0.35 | 1.2 | 9.84 ± 0.99 | 11.3 ± 0.64 | 1.1 | 2.81 ± 0.29 | 6.96 ± 1.11 | 2.5 |
| Small gut | 14.2 ± 1.30 | 17.5 ± 1.08 | 1.2 | 14.6 ± 0.57 | 18.7 ± 2.94 | 1.3 | 3.56 ± 0.27 | 13.0 ± 1.31 | 3.7 |
| Stomach | 7.68 ± 0.64 | 7.21 ± 1.51 | 0.9 | 7.40 ± 1.56 | 11.2 ± 1.20 | 1.5 | 3.25 ± 0.44 | 5.82 ± 0.35 | 1.8 |
| Liver | 20.6 ± 8.86 | 44.7 ± 11.3 | 2.2 | 10.2 ± 0.83 | 45.3 ± 5.58 | 4.5 | 1.69 ± 0.16 | 13.6 ± 1.05 | 8.1 |
| Kidney | 36.2 ± 4.70 | 33.2 ± 1.86 | 0.9 | 22.3 ± 1.36 | 21.6 ± 1.30 | 1.0 | 5.68 ± 0.44 | 9.05 ± 0.39 | 1.6 |
| Lung | 23.3 ± 1.31 | 25.3 ± 1.79 | 1.1 | 23.4 ± 1.50 | 24.5 ± 2.72 | 1.0 | 5.05 ± 0.58 | 18.9 ± 0.87 | 3.8 |
| Spleen | 16.6 ± 0.80 | 18.9 ± 1.43 | 1.1 | 23.7 ± 1.09 | 22.5 ± 0.70 | 0.9 | 16.6 ± 1.04 | 29.2 ± 1.83 | 1.8 |
| Heart | 21.5 ± 1.87 | 23.6 ± 1.45 | 1.1 | 12.6 ± 0.75 | 13.7 ± 0.78 | 1.1 | 1.69 ± 0.14 | 4.74 ± 0.39 | 2.8 |
| Ovary | 7.41 ± 0.50 | 9.58 ± 0.77 | 1.3 | 6.74 ± 0.32 | 9.15 ± 1.11 | 1.4 | 4.76 ± 1.10 | 5.85 ± 1.06 | 1.2 |
| Uterus | 7.98 ± 1.92 | 10.6 ± 1.10 | 1.3 | 12.1 ± 0.48 | 8.07 ± 1.13 | 0.7 | 6.78 ± 1.98 | 12.9 ± 1.52 | 1.9 |
| Breast | 5.71 ± 0.53 | 6.46 ± 0.43 | 1.1 | 4.25 ± 0.60 | 7.11 ± 1.06 | 1.7 | 1.47 ± 0.22 | 5.42 ± 0.63 | 3.7 |

Data, means ± standard errors in nmol g$^{-1}$ tissue (for plasma, in nM); ratio, drug concentration in *mdr1a(–/–)* vs wild-type mice.

 *© Cancer Research Campaign 1999*

**Table 3** Tissue levels of doxorubicinol, 7-deoxydoxorubicinolone and 7-deoxydoxorubicinone at 1, 4 and 24 h after i.v. administration of 5 mg $kg^{-1}$ doxorubicin to wild-type and *mdr1a(–/–)* mice

| **Metabolite** | **Time (h)** | **Heart** | | | **Liver** | | | **Kidney** | | |
|---|---|---|---|---|---|---|---|---|---|---|
| | | **Wild-type** | ***mdr1a(–/–)*** | **Ratio** | **Wild-type** | ***mdr1a(–/–)*** | **Ratio** | **Wild-type** | ***mdr1a(–/–)*** | **Ratio** |
| Doxorubicinol | 1 | 0.14 ± 0.01 | 0.15 ± 0.01 | 1.1 | 0.21 ± 0.09 | 0.69 ± 0.20 | 3.3 | 0.46 ± 0.10 | 0.45 ± 0.06 | 1.0 |
| | 4 | 0.21 ± 0.01 | 0.16* ± 0.01 | 0.8 | 0.19 ± 0.04 | 1.53* ± 0.14 | 8.1 | 0.33 ± 0.05 | 0.48 ± 0.07 | 1.5 |
| | 24 | 0.04 ± 0.00 | 0.09* ± 0.01 | 2.3 | 0.05 ± 0.01 | 0.66* ± 0.06 | 13.8 | 0.08 ± 0.01 | 0.22* ± 0.04 | 2.8 |
| 7-Deoxydoxorubicinolone | 1 | 0.35 ± 0.10 | 0.22 ± 0.04 | 0.6 | 16.8 ± 5.55 | 14.3 ± 3.17 | 0.9 | 2.86 ± 0.73 | 3.13 ± 0.54 | 1.1 |
| | 4 | 0.09 ± 0.01 | 0.09 ± 0.02 | 1.1 | 1.99 ± 0.52 | 9.84 ± 3.26 | 4.9 | 0.62 ± 0.06 | 0.65 ± 0.09 | 1.1 |
| | 24 | <LLQ[a] | 0.05 ± 0.00 | | 0.10 ± 0.01 | 0.40* ± 0.02 | 4.3 | 0.05 ± 0.01 | 0.16* ± 0.01 | 3.5 |
| 7-Deoxydoxorubicinone | 1 | <LLQ[a] | <LLQ[a] | | 1.54 ± 0.15 | 2.80* ± 0.44 | 1.8 | 0.56 ± 0.18 | 0.60 ± 0.07 | 1.1 |
| | 4 | <LLQ[a] | <LLQ[a] | | 0.46 ± 0.12 | 1.65* ± 0.37 | 3.6 | 0.09 ± 0.01 | 0.15* ± 0.02 | 1.6 |
| | 24 | <LLQ[a] | <LLQ[a] | | 0.03 ± 0.00 | 0.15* ± 0.01 | 4.4 | <LLQ[a] | <LLQ[a] | |

Data, means ± standard errors in nmol $g^{-1}$ tissue; ratio, drug concentration in *mdr1a(–/–)* vs wild-type mice. [a]<LLQ, concentration below the lower limit of quantification of the analytical assay; *$P$<0.05 vs wild-type mice.

**Table 4** Excretion of doxorubicin and metabolites within 96 h after i.v. administration of 5 mg $kg^{-1}$ doxorubicin to wild-type and *mdr1a(–/–)* mice

| **Compound** | **Faeces** | | **Urine** | |
|---|---|---|---|---|
| | **Wild-type** | ***mdr1a(–/–)*** | **Wild-type** | ***mdr1a(–/–)*** |
| Doxorubicin | 5.2 ± 0.3 | 4.1 ± 0.5 | 10.9 ± 0.7 | 15.4* ± 1.1 |
| Doxorubicinol | 0.2 ± 0.0 | 0.4* ± 0.0 | 1.4 ± 0.2 | 2.0 ± 0.2 |
| 7-Deoxydoxorubicinolone | 2.2 ± 0.2 | 1.7* ± 0.1 | <0.1 | 0.1 ± 0.1 |
| 7-Deoxydoxorubicinone | 5.2 ± 0.5 | 3.1* ± 0.3 | 0.5 ± 0.1 | 0.2* ± 0.0 |

Data, means ± standard errors as percentage of the administered dose; *$P$<0.05 vs wild-type mice.

### DISCUSSION

The most remarkable observation of the present study is the increased accumulation of doxorubicin in heart, brain and liver of *mdr1a(–/–)* mice compared with wild-type mice. No or small differences in drug accumulation were observed for most other tissues. In addition, the absence of mdr1a P-glycoprotein resulted in only slight alterations of the plasma pharmacokinetics and excretion of doxorubicin and metabolites. These results are in sharp contrast to those of similar studies with vinblastine and paclitaxel (Van Asperen et al, 1996; Sparreboom et al, 1997). This indicates either that mdr1a P-glycoprotein has a minor impact on the pharmacokinetics of doxorubicin in comparison with vinblastine or paclitaxel, or that the absence of this protein is more efficiently compensated by alternative mechanisms of drug elimination for doxorubicin than for the other drugs.

A relatively small impact of mdr1a P-glycoprotein on the plasma pharmacokinetics of doxorubicin may be explained by several factors. For example, mdr1a P-glycoprotein may have a low affinity for doxorubicin. In comparison with non-P-glycoprotein-expressing parental cells, the resistance of *mdr1a* transfected cells to doxorubicin and vinblastine was shown to be 35- and 53-fold higher respectively (Tang-Wai et al, 1995). Although this suggests that vinblastine may be somewhat more efficiently transported by mdr1a P-glycoprotein than doxorubicin, the latter still seems to be a rather good substrate. An alternative explanation may be the difference in excretion of unchanged drug. Excretion studies with vinblastine (over 48 h) and paclitaxel (over 96 h) demonstrated that 24% and 40% of the i.v. administered dose, respectively, was excreted unchanged in the faeces of wild-type mice, whereas this was reduced to 10% and 2% in *mdr1a(–/–)* mice respectively (Van Asperen et al, 1996; Sparreboom et al, 1997). This indicates that mdr1a P-glycoprotein in the gut wall and/or in the liver substantially contributes to the elimination of vinblastine and paclitaxel because the urinary excretion of these compounds was similar in both types of mice. Furthermore, a comparable biliary excretion of unchanged paclitaxel was observed in *mdr1a(–/–)* and wild-type mice (Sparreboom et al, 1997) suggesting that the diminished clearance of paclitaxel in the former was caused by the absence of intestinal P-glycoprotein, e.g. as a result of a substantially enhanced reuptake of unchanged drug from the intestinal lumen. The reduced clearance of vinblastine may be explained analogously. However, within 96 h after administration of doxorubicin, only 5% of the dose was excreted unchanged in faeces of wild-type mice, indicating that any possible reuptake from the intestinal lumen in *mdr1a(–/–)* mice is of minor importance for the overall clearance. Furthermore, it is also possible that the elimination of doxorubicin is mainly mediated by transport mechanisms other than P-glycoprotein.

Our previous data on vinblastine suggested that an increased drug accumulation in most tissues of *mdr1a(–/–)* mice compared with wild-type mice may be caused by the concurrently higher plasma levels (Van Asperen et al, 1996). Possibly, the same holds true for other P-glycoprotein substrate drugs. Hence, a minor impact of mdr1a P-glycoprotein on the accumulation of doxorubicin in most tissues may result from a small difference in plasma pharmacokinetics between *mdr1a(–/–)* and wild-type mice.

The tissue distribution of doxorubicin is previously examined in mice treated in combination with GF120918, cyclosporin A or SDZ PSC 833 (Hyafil et al, 1993; Colombo et al, 1994; Bellamy et al, 1995; Gonzalez et al, 1995). Co-administration of these reversal agents did only slightly change the tissue distribution of doxorubicin, which corresponds with the results of our experiments in *mdr1a(–/–)* mice. The impact of reversal agents on the plasma pharmacokinetics of doxorubicin has also been investigated (Hyafil et al, 1993; Colombo et al, 1994; Gonzalez et al, 1995; Dantzig et al, 1996). Small differences in the plasma pharmacokinetics of doxorubicin were observed between control mice and mice co-treated with GF120918, cyclosporin A (formulated in olive oil), SDZ PSC 833, LY335979 or verapamil, which also corresponds

© Cancer Research Campaign 1999

with the presently observed differences between wild-type and *mdr1a(–/–)* mice. Treatment with cyclosporin A (formulated in Cremophor EL), however, resulted in a substantially increased AUC of doxorubicin (Dantzig et al, 1996), but this was probably caused by the vehicle Cremophor EL as Webster et al (1996) clearly demonstrated that this compound alters the plasma pharmacokinetics of doxorubicin. Despite minor pharmacokinetic alterations, co-administration of the reversal agents cyclosporin A or SDZ PSC 833 resulted in a high mortality of non-tumour-bearing mice treated with doxorubicin, whereas each of these compounds alone was well tolerated (Colombo et al, 1994; Bellamy et al, 1995; Gonzalez et al, 1995). The increased toxicity has been suggested to be, at least partially, caused by an increased accumulation of doxorubicin in the heart because in this tissue up to twofold higher drug concentrations and severe myocardial damage were observed (Bellamy et al, 1995). Several studies have shown that not only doxorubicin itself but also its metabolite doxorubicinol has cardiotoxic properties (Olson et al, 1988; De Jong et al, 1993). Our findings support the idea that a blockade of P-glycoprotein may enhance the risk of cardiac toxicity upon treatment with doxorubicin because the absolute concentrations of both doxorubicin and doxorubicinol were more than twofold higher in the hearts of *mdr1a(–/–)* mice at 24 h after drug administration (Tables 2 and 3). As significant amounts of mdr1b P-glycoprotein are still present in the hearts of these animals, even more pronounced effects may be expected upon a complete blockade of drug-transporting P-glycoproteins.

For the clinical situation, these data suggest that a blockade of endogenous P-glycoprotein, for example by a reversal agent, may increase the risk of cardiotoxicity when patients are co-treated with doxorubicin. In general, an increased cardiotoxicity of doxorubicin has hitherto not been observed in clinical trials with reversal agents (Erlichman et al, 1993; Bartlett et al, 1994; Giaccone et al, 1997), but this may be explained by the insufficient potency of the currently available reversal agents to block endogenous P-glycoprotein completely under in vivo conditions. Furthermore, it is important to stress that reversal agents also frequently cause other effects beyond those resulting from inhibition of P-glycoprotein. For example, SDZ PSC 833 has been reported to inhibit the biliary elimination of [$^3$H]digoxin in *mdr1a/1b(–/–)* mice (Mayer et al, 1997). The investigators demonstrated that this was not the result of cholestasis, and they suggested that SDZ PSC 833 does not only inhibit P-glycoprotein but also another hepatic transporter (or transporters) of [$^3$H]digoxin. Furthermore, metabolic inhibition can easily occur because many reversal agents and anti-cancer drugs are substrates for the cytochrome P450 3A enzymes (Wacher et al, 1995).

In conclusion, only slight alterations in the plasma pharmacokinetics of doxorubicin were found in the absence of mdr1a P-glycoprotein. However, a substantially prolonged presence of both doxorubicin and doxorubicinol was observed in the hearts of *mdr1a(–/–)* mice, which suggests that a blockade of endogenous P-glycoprotein in patients, for example by the clinical application of a reversal agent, may enhance the risk of cardiotoxicity upon co-administration of doxorubicin.

### REFERENCES

Bartlett NL, Lum BL, Fisher GA, Brophy NA, Ehsan MN, Halsey J and Sikic BI (1994) Phase I trial of doxorubicin with cyclosporine as a modulator of multidrug resistance. *J Clin Oncol* **12**: 835–842

Bellamy WT, Peng YM, Odeleye A, Ellsworth L, Xu MJ, Grogan TM and Weinstein RS (1995) Cardiotoxicity in the SCID mouse following administration of doxorubicin and cyclosporin A. *Anti-Cancer Drugs* **6**: 736–743

Colombo T, Zucchetti M and D'Incalci M (1994) Cyclosporin A markedly changes the distribution of doxorubicin in mice and rats. *J Pharmacol Exp Ther* **269**: 22–27

Cordon-Cardo C, O'Brien JP, Casals D, Rittman-Grauer L, Biedler JL, Melamed MR and Bertino JR (1989) Multidrug-resistance gene (P-glycoprotein) is expressed by endothelial cells at blood–brain barrier sites. *Proc Natl Acad Sci USA* **86**: 695–698

Croop JM, Raymond M, Haber D, Devault A, Arceci RJ, Gros P and Housman DE (1989) The three mouse multidrug resistance (*mdr*) genes are expressed in a tissue-specific manner in normal mouse tissues. *Mol Cell Biol* **9**: 1346–1350

Dantzig AH, Shepard RL, Cao J, Law KL, Ehlhardt WJ, Baughman TM, Bumol TF and Starling JJ (1996) Reversal of P-glycoprotein-mediated multidrug resistance by a potent cyclopropyldibenzosuberane modulator, LY335979. *Cancer Res* **56**: 4171–4179

De Jong J, Schoofs PR, Snabilié AM, Bast A and van der Vijgh WJF (1993) The role of biotransformation in anthracycline-induced cardiotoxicity in mice. *J Pharmacol Exp Ther* **266**: 1312–1320

Endicott JA and Ling V (1989) The biochemistry of P-glycoprotein-mediated multidrug resistance. *Annu Rev Biochem* **58**: 137–171

Erlichman C, Moore M, Thiessen JJ, Kerr IG, Walker S, Goodman Ph, Bjarnason G, DeAngelis C and Bunting P (1993) Phase I pharmacokinetic study of cyclosporin A combined with doxorubicin. *Cancer Res* **53**: 4837–4842

Giaccone G, Linn SC, Welink J, Catimel G, Stieltjes H, van der Vijgh WJF, Eeltink C, Vermorken JB and Pinedo HM (1997) A dose-finding and pharmacokinetic study of reversal of multidrug resistance with SDZ PSC 833 in combination with doxorubicin in patients with solid tumors. *Clin Cancer Res* **3**: 2005–2015

Gonzalez O, Colombo T, De Fusco M, Imperatori L, Zucchetti M and D'Incalci M (1995) Changes in doxorubicin distribution and toxicity in mice pretreated with the cyclosporin analogue SDZ PSC 833. *Cancer Chemother Pharmacol* **36**: 335–340

Hyafil F, Vergely C, du Vignaud P and Grand-Perret T (1993) In vitro and in vivo reversal of multidrug resistance by GF120918, an acridonecarboxamide derivative. *Cancer Res* **53**: 4595–4602

Mayer U, Wagenaar E, Beijnen JH, Smit JW, Meijer DKF, van Asperen J, Borst P and Schinkel AH (1996) Substantial excretion of digoxin via the intestinal mucosa and prevention of long-term digoxin accumulation in the brain by mdr1a P-glycoprotein. *Br J Pharmacol* **119**: 1038–1044

Mayer U, Wagenaar E, Dorobek B, Beijnen JH, Borst P and Schinkel AH (1997) Full blockade of intestinal P-glycoprotein and extensive inhibition of blood–brain barrier P-glycoprotein by oral treatment of mice with PSC833. *J Clin Invest* **100**: 2430–2436

Mross K, Maessen P, van der Vijgh WJF, Gall H, Boven E and Pinedo HM (1988) Pharmacokinetics and metabolism of epidoxorubicin and doxorubicin in humans. *J Clin Oncol* **6**: 517–526

Olson RD, Mushlin PS, Brenner DE, Fleischer S, Cusack BJ, Chang BK and Boucek RJ (1988) Doxorubicin cardiotoxicity may be caused by its metabolite, doxorubicinol. *Proc Natl Acad Sci USA* **85**: 3585–3589

Schinkel AH, Smit JJM, van Tellingen O, Beijnen JH, Wagenaar E, van Deemter L, Mol CAAM, van der Valk MA, Robanus-Maandag EC, te Riele HPJ, Berns AJM and Borst P (1994) Disruption of the mouse *mdr1a* P-glycoprotein gene leads to a deficiency in the blood–brain barrier and to increased sensitivity to drugs. *Cell* **77**: 491–502

Schinkel AH, Wagenaar E, van Deemter L, Mol CAAM and Borst P (1995) Absence of the mdr1a P-glycoprotein in mice affects tissue distribution and pharmacokinetics of dexamethasone, digoxin, and cyclosporin A. *J Clin Invest* **96**: 1698–1705

Schinkel AH, Wagenaar E, Mol CAAM and van Deemter L (1996) P-glycoprotein in blood–brain barrier of mice influences the brain penetration and pharmacological activity of many drugs. *J Clin Invest* **97**: 2517–2524

Schinkel AH, Mayer U, Wagenaar E, Mol CAAM, van Deemter L, Smit JJM, van der Valk MA, Voordouw AC, Spits H, van Tellingen O, Zijlmans JMJM, Fibbe WE and Borst P (1997) Normal viability and altered pharmacokinetics in mice lacking mdr1-type (drug-transporting) P-glycoproteins. *Proc Natl Acad Sci USA* **94**: 4028–4033

Sparreboom A, van Asperen J, Mayer U, Schinkel AH, Smit JW, Meijer DKF, Borst P, Nooijen WJ, Beijnen JH and van Tellingen O (1997) Limited oral bioavailability and active epithelial excretion of paclitaxel (Taxol) caused by P-glycoprotein in the intestine. *Proc Natl Acad Sci USA* **94**: 2031–2035

Tang-Wai DF, Kajiji S, DiCapua F, de Graaf D, Roninson IB and Gros P (1995) Human (*MDR1*) and mouse (*mdr1, mdr3*) P-glycoproteins can be distinguished

 *© Cancer Research Campaign 1999*

by their respective drug resistance profiles and sensitivity to modulators. *Biochemistry* **34**: 32–39

Thiebaut F, Tsuruo T, Hamada H, Gottesman MM, Pastan I and Willingham MC (1987) Cellular localization of the multidrug resistance gene product P-glycoprotein in normal human tissues. *Proc Natl Acad Sci USA* **84**: 7735–7738

Tsuruo T, Iida H, Tsukagoshi S and Sakurai Y (1981) Overcoming of vincristine resistance in P388 leukemia in vivo and in vitro through enhanced cytotoxicity of vincristine and vinblastine by verapamil. *Cancer Res* **41**: 1967–1972

Van Asperen J, Schinkel AH, Beijnen JH, Nooijen WJ, Borst P and van Tellingen O (1996) Altered pharmacokinetics of vinblastine in mdr1a P-glycoprotein-deficient mice. *J Natl Cancer Inst* **88**: 994–999

Van Asperen J, van Tellingen O and Beijnen JH (1998) Determination of doxorubicin and metabolites in murine specimens by high-performance liquid chromatography. *J Chromatogr B* **712**: 129–143

Wacher VJ, Wu C-Y and Benet LZ (1995) Overlapping substrate specificities and tissue distribution of cytochrome P450 3A and P-glycoprotein: implications for drug delivery and activity in cancer chemotherapy. *Mol Carcinogen* **13**: 129–134

Webster LK, Cosson EJ, Stokes KH and Millward MJ (1996) Effect of the paclitaxel vehicle, Cremophor EL, on the pharmacokinetics of doxorubicin and doxorubicinol in mice. *Br J Cancer* **73**: 522–524

*© Cancer Research Campaign 1999*
